# Editorial: Infectious disease agents and cancer

**DOI:** 10.3389/fcell.2024.1388423

**Published:** 2024-03-12

**Authors:** Ana Igea, Océane C. B. Martin, Tomer Cooks, Ioannis S. Pateras

**Affiliations:** ^1^ Instituto de Investigación Sanitaria de Santiago de Compostela (IDIS), Santiago de Compostela, Spain; ^2^ Mobile Genomes, Centre for Research in Molecular Medicine, and Chronic Diseases (CiMUS), University of Santiago de Compostela (USC), Santiago de Compostela, Spain; ^3^ Biological and Medical Sciences Department, University Bordeaux, Centre National de la Recherche Scientifique (CNRS), Institut de Biochimie et Génétique Cellulaires (IBGC), Unité Mixte de Recherche (UMR) 5095, Bordeaux, France; ^4^ The Shraga Segal Department of Microbiology, Immunology and Genetics, Ben-Gurion University of the Negev, Beer-Sheva, Israel; ^5^ Second Department of Pathology, “Attikon” University Hospital, Medical School, National and Kapodistrian University of Athens, Athens, Greece

**Keywords:** infection, infectious agents, cancer, virus, bacteria, parasites, inflammation, immune response

The link between infectious disease agents and cancer dates back to early 20th century, often challenging prevailing dogmas regarding human tumorigenesis. It reflects a complex interplay between the infectious agents and the host that is understudied. This Research Topic includes original research and review papers that shed light on different aspects of this association.

The role of parasitic infections in cancer development is exemplified by liver flukes, where infection with *Opisthorchis viverrine*, *Clonorchis sinensis* and *Schistomomiasis japonica* is associated with increased risk for cholangiocarcinoma (van Tong et al., 2017). In the review paper by Kaufman et al*.* it is demonstrated the pro- and anti-tumorigenic role of the protozoan *Trypanosoma Cruzi*, the causing agent of Chagas disease. Experimental evidence depict contradictory findings, although it seems that most studies support an anti-tumor potential including enhancement of tumor immunogenicity and inhibition of invasion and metastasis. The dual role of *Trypanosoma Cruzi* infection in carcinogenesis could be attributed to the presence of different parasitic strains and the tumor types analyzed. Further studies are required to unveil the association of *Trypanosoma Cruzi* infection with different types of cancer.

The causative agent of HIV-associated Kaposi’s sarcoma (KS) is a double-stranded DNA virus called Kaposi sarcoma associated herpesvirus (KSHV) (also know as human herpesvirus-8, HHV-8), and usually arises in the context of HIV-infected patients. During the early AIDS epidemic, a significant percentage of patients with HIV developed KS, however after the introduction of antiretroviral therapy its incidence has decreased (Goncalves et al., 2017). Compromised T cell immune response allows KSHV-infected cells to thrive (Robey et al., 2010). Within this context, Clutton et al. demonstrated that a subset of T lymphocytes in HIV patients with KS demonstrated low CD8 surface (denoted as CD8^dim^) status along with upregulation of the senescence-associated marker CD57. These CD8^dim^CD57+ T cells expressed lower levels of Peroxisome proliferator-activated receptor-gamma coactivator (PGC)-1alpha (PGC1α) the master regulator of mitochondrial biogenesis and exhibited reduced mitochondrial respiration compared to CD8^bright^ T cells. This study reveals a novel immunophenotypic profile of T cells in this setting, which warrants further examination. For instance, it is important to address the antigen specificity of CD8^dim^CD57+ T cells and whether they are involved in the pathogenesis of KS in HIV.

Human papilloma viruses (HPV) are double-stranded DNA viruses that infect basal epithelial cells resulting to benign and malignant neoplasms in anogenital tract and head and neck (de Martel et al., 2017). Impairment of immune system in HPV-infected individuals promotes cancer development (Zhou et al., 2019). However, the exact mechanisms involved in the crosstalk between HPV and host immune response is not clarified. To further shed light in this issue, Leventakou et al. assessed the expression of hsa-miR-20a-5p, hsa-miR-106b-5p, hsa-miR-200a-3p and has-miR125b-5p in cervical intraepithelial neoplasia and cervical carcinomas from 115 patients with well-characterized HPV status. The selection of these miRNAs was based on computational analysis that predicted that these miRNAs potentially target the mRNA of the immune checkpoint inhibitor *Programmed Death—Ligand 1* (*PD-L1*). The authors demonstrated that hsa-miR-20a-5p and hsa-miR-106b-5p were overexpressed in high-grade lesions. As *PD-L1* mRNA expression was elevated with the lesion progression, these findings are not in accordance with the hypothesis, suggesting that these miRNAs should be considered as oncomiRs. On the other hand, a slight decrease in the hsa-miR-125b-5p status in full blown cancer verifies the initial hypothesis. Functional studies are required to verify the link of these miRNAs with PD-L1 as well as its role in cervical carcinogenesis. Yu et al. reviewed the current status of immunotherapy in HPV-dependent and HPV-independent head and neck squamous cell carcinoma (HNSCC) patients. Accumulating knowledge of immune response during head and neck carcinogenesis has allowed the implementation of several immune checkpoint inhibitors including antibodies targeting PD-L1, Programmed Death 1 (PD-1), Cytotoxic T-lymphocyte-associated protein-4 (CTLA-4), Lymphocyte activation gene 3 protein (LAG3) and the introduction of costimulatory agonists like CD40 in HNSCC treatment. Notably combinational of different immune-based therapies complementing radio- and chemo-therapy could improve therapeutic outcome, although the identification of safe regimens with minimal side-effects is necessary. Besides, even though the application of therapeutic vaccines in HPV(+) and HPV(−) cases is still limited, existing data are promising, suggesting a brighter future for HNSCC treatment.

Huang and He studied the role of Basigin (BSG) in cancer. BSG is a transmembrane glycoprotein belonging to the immunoglobulin superfamily (also known as CD147) and serves as target that allows SAR-CoV-2 to enter host cells. From another perspective BSG is implicated in the development of multiple cancers. In this study, the authors assessed the status and clinical role of BSG in multiple cancers. BSG was overexpressed in 14 cancers including bladder carcinoma, invasive breast carcinoma, stomach adenocarcinoma, thyroid carcinoma, and uterine corpus endometrial carcinoma, while downregulated in colorectal adenocarcinoma. Interestingly, BSG expression could serve as a prognostic marker in certain cancers. Besides, this study revealed the association of BSG expression with Tumor Mutational Burden (TMB), mismatch repair protein (MMR) status and microsatellite instability (MSI) suggesting a potential link of BSG with immune response.

Overall, these studies stress out the complexity in microbe—host interaction. Although the underlying mechanisms involved in infection persistence and host immunity are understudied, it is clear that defective immunity promotes cancer. Besides, a critical factor is tissue damage during chronic inflammation, which provides the fertile soil for cancer development (Pateras et al., 2024). Furthermore, apart from epidemiological data linking several cancers with chronic inflammation due to persistent infections, accumulating evidence support a direct etiological role of common infectious agents including bacteria in cancer development (Hansen et al., 2021). Along this line, we recently demonstrated how genotoxin-producing *Salmonella enterica* exerts an immunomodulatory role in the intestine but not in the liver, stressing out the relevance of the tissue microenvironment (Lopez Chiloeches et al., 2023). In 2022 D. Hanahan in the “Hallmarks of Cancer: New Dimensions” incorporated the term “polymorphic microbiomes” as an enabling characteristic, highlighting the role of the microbiome in the acquisition of cancer hallmarks (Hanahan, 2022). We are clearly at the beginning of a fascinating era in capturing the host–microbiome interplay.
